# Rapid production of human liver scaffolds for functional tissue engineering by high shear stress oscillation-decellularization

**DOI:** 10.1038/s41598-017-05134-1

**Published:** 2017-07-17

**Authors:** Giuseppe Mazza, Walid Al-Akkad, Andrea Telese, Lisa Longato, Luca Urbani, Benjamin Robinson, Andrew Hall, Kenny Kong, Luca Frenguelli, Giusi Marrone, Oliver Willacy, Mohsen Shaeri, Alan Burns, Massimo Malago, Janet Gilbertson, Nigel Rendell, Kevin Moore, David Hughes, Ioan Notingher, Gavin Jell, Armando Del Rio Hernandez, Paolo De Coppi, Krista Rombouts, Massimo Pinzani

**Affiliations:** 1 UCL Institute for Liver and Digestive Health, Royal Free Hospital. University College London, London, UK; 2 Stem Cells and Regenerative Medicine Section, Developmental Biology and Cancer Programme, UCL Great Ormond Street Institute for Child Health. University College London, London, UK; 3 Department of Bioengineering, Cellular and Molecular Biomechanics. Imperial College, London, UK; 4 School of Physics and Astronomy, University of Nottingham, Nottingham, UK; 5 CN Bio Innovations Limited. BioPark Hertfordshire, Broadwater Road, Welwyn Garden City, Hertfordshire, UK; 6 Department of Clinical Genetics, Erasmus Medical Centre, Rotterdam, Netherlands; 7 Wolfson Drug Discovery Unit, Centre for Amyloidosis and Acute Phase Proteins, Royal Free Hospital. University College London, London, UK; 8 Center for Nanotechnology and Regenerative Medicine, Division of Surgery and Interventional Science. University College London, London, UK

## Abstract

The development of human liver scaffolds retaining their 3-dimensional structure and extra-cellular matrix (ECM) composition is essential for the advancement of liver tissue engineering. We report the design and validation of a new methodology for the rapid and accurate production of human acellular liver tissue cubes (ALTCs) using normal liver tissue unsuitable for transplantation. The application of high shear stress is a key methodological determinant accelerating the process of tissue decellularization while maintaining ECM protein composition, 3D-architecture and physico-chemical properties of the native tissue. ALTCs were engineered with human parenchymal and non-parenchymal liver cell lines (HepG2 and LX2 cells, respectively), human umbilical vein endothelial cells (HUVEC), as well as primary human hepatocytes and hepatic stellate cells. Both parenchymal and non-parenchymal liver cells grown in ALTCs exhibited markedly different gene expression when compared to standard 2D cell cultures. Remarkably, HUVEC cells naturally migrated in the ECM scaffold and spontaneously repopulated the lining of decellularized vessels. The metabolic function and protein synthesis of engineered liver scaffolds with human primary hepatocytes reseeded under dynamic conditions were maintained. These results provide a solid basis for the establishment of effective protocols aimed at recreating human liver tissue *in vitro*.

## Introduction

The development and functionalization of biomaterials have been actively pursued to enable more effective applications of tissue engineering. In particular, the development of 3D-biological scaffolds, based on a natural extracellular matrix (ECM) protein structure, has been shown to provide a more physiological platform for cell engraftment and function^1–4^. In addition, the understanding of human physiological and pathophysiological conditions could be improved by employing appropriate 3D-ECM microenvironments reflecting the complexity of normal and diseased tissues, respectively^5–9^.

The main challenge of liver tissue engineering is to recapitulate the complexity of human liver micro-architecture. The hepatic ECM is a complex network of macromolecules that not only provides cells with an extracellular scaffold but also plays an important role in the regulation of cellular functions^10, 11^. The regulation of key cellular features, such as differentiation and motility, is modulated by biochemical signals as well as by the surrounding physical environment^12–15^. Therefore, biomaterials should be able to reproduce the essential characteristics of the physiological extracellular scaffold including tissue-specific ECM protein composition, 3D-microarchitecture, stiffness and pro-angiogenic properties in order to support cellular growth and maintain cell phenotypes.

Decellularization is a process that removes cellular and immunogenic materials from tissues and organs while maintaining the mechanical and bioactive properties of the tissue^16^. This provides the optimal micro-environment for the repopulation with organ-specific cell types leading to the development of engineered tissues and organs. Accordingly, major advances in the decellularization-recellularization technology have been achieved for the development of whole engineered organs due to their attractive application in the area of whole organ transplantation^1, 2, 17–21^.

Along these lines, we have recently reported the development of whole acellular human liver scaffolds obtained by the decellularization of human livers unsuitable for transplantation and we have addressed its biocompatibility and the suitability to be repopulated with different types of human liver cells^22^.

Despite the progress achieved in establishing novel and improved protocols for the development of whole organ scaffolds, no methodological advancement has been reported for the decellularization of tissue sections. Importantly, the development of small scale acellular scaffolds could provide tissue-specific 3D-ECM platforms to be used as an alternative to standard 2D-cell cultures on artificial materials such as plastic. This will allow the introduction of *in vitro* experimental conditions much closer to human physiology and pathology, and provide a framework to investigate drug action and metabolism.

The main aim of the present study was to develop and fully characterise a novel methodology for improving the generation of small size 3D-ECM scaffold from sections of healthy human livers. This methodology, based on high *g*-force oscillation/high shear stress and markedly reduced processing time, represents an alternative to the established agitation-decellularization procedures so far reported^23, 24^. These procedures were characterized by lengthy processes and prolonged exposure to detergents^22, 23, 25^.

Here, we demonstrate a rapid and efficient production of human acellular liver tissue cubes (ALTCs) with preserved ECM protein composition, 3D-architecture, angiogenic potential, topography and stiffness, allowing tissue engineering with different types of human hepatic cells. Overall, the work presented herein is a key advance for the development of *in vitro* human 3D-platforms for investigating disease pathophysiology, pharmacological target discovery, drug toxicity assessment as well as engineered tissue transplant.

## Results

### Optimization of the agitation-decellularization procedure of human acellular liver tissue cubes

The procedure was carried out by employing a stepwise protocol using increasing g-force intensity in order to remove immunogenic cellular materials while preserving ECM proteins as well as reducing processing time and exposure to detergents. Therefore, the first step was to define the *g*-force value to be employed during agitation-decellularization and apply this value consistently through the different protocols.

### Determining g- force

The agitation systems were characterized by different agitation speeds defined by different g-force values. The *g*-force, which refers to the relative centrifuge force (RCF), is expressed as REF = 1.118 × r × (rpm)^2^ and was adopted to estimate the *g*-force value employed in previously published protocols using either an orbital shaker or a magnetic stirrer^25–30^.

The novel system designed for decellularizing biological tissue considers a tissue sample undergoing harmonic motion characterized by a frequency *f* Hz and amplitude *Û* m and assuming it starts at zero displacement (*i.e., x* = 0 and *t* = 0) will occupy the position $x=\hat{{\rm{U}}}sin(f2\pi t)$ (considering the difference in density between the tissue and it surrounding solution is negligible). Furthermore, a particle (or tissue in this case) which moves under simple harmonic motion will be represented by the equation *x*” = −*w*2*x*, where *w* = 2*πf*. Reformulating these equation according to Newton’s second law, Force (*F*) equals mass (*m*) multiplied by acceleration (*a*) [*F* = *ma*], results in the equation $\,F\,=\,-m[(2\pi f)2]\,\hat{{\rm{U}}}sin(f2\pi t)$. This is greatest when |sin| = 1. Therefore, it will be at its maximum/minimum when $F=+/-\hat{{\rm{U}}}m[(2\pi f)2]$. Finally, by dividing the equation by the force experienced by gravity (*mg*), the equation for *g* $-force\,{\rm{is}}=\,(\hat{\rm U}/g)\ast [(2\pi f)2]$.

### Protocol optimization

The general study protocol followed the scheme illustrated in Supplementary Fig. 1a.

Initial experiments illustrated in Supplementary Fig. 1b and c employed low *g*-force intensity (0.4 *g*). After 8 and 16 days of agitation with an orbital shaker (OS), the resultant tissue appeared macroscopically brownish indicating incomplete decellularization (Supplementary Table 1a; protocol OS1 and OS2). Histological examination employing hematoxylin and eosin (H&E) and Sirius Red (SR) staining revealed the presence of cellular material within the tissue (Supplementary Fig. 1b,c). Although, DNA quantification showed a significant reduction in the total amount of DNA after the procedure (p < 0.0001; Supplementary Fig. 1e) this was still above the accepted threshold of 50 ng/mg of tissue^16^.

Subsequently, *g*-force was increased to 5–9 *g* obtained by agitating the tissue with a magnetic stirrer (MS) (Supplementary Table 1a; protocol MS). After 8 days of agitation the tissue became translucent (Supplementary Fig. 1d) and histological staining confirmed preservation of collagen and elastin filaments, while nuclear materials were removed. This latter finding was confirmed by DNA quantification which showed significant reduction of DNA (p < 0.0001) but the amount of DNA was slightly above 50 ng/mg threshold (Supplementary Fig. 1e).

**Figure 1:**
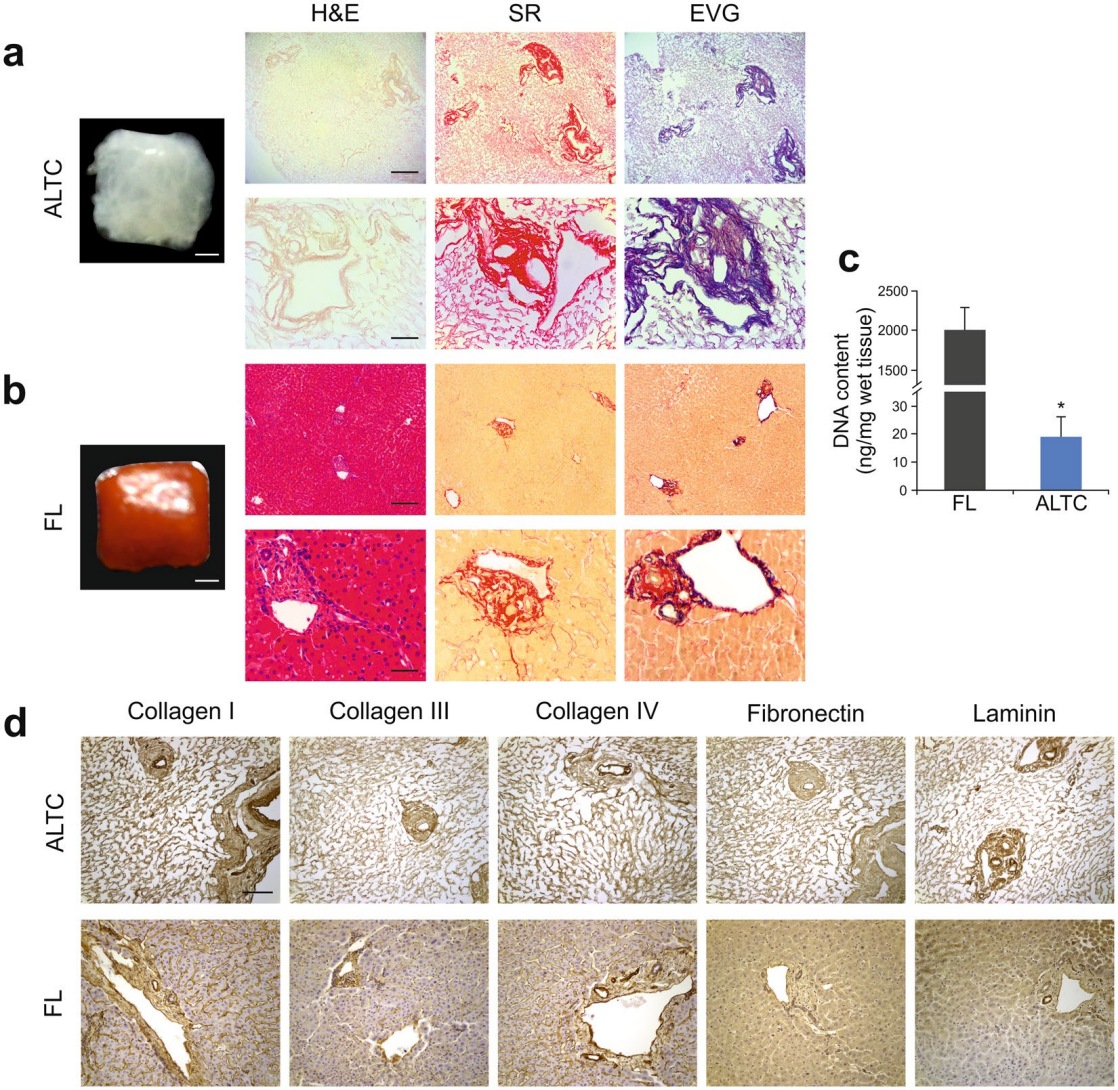
Decellularization of human liver cubes. (**a**) Macroscopic appearance and histological analysis after decellularization, confirming elimination of nuclear (blue; H&E) and cellular material (yellow; SR) and preservation of collagen (red; SR) and elastin (blue/black; EVG). (**b**) Macroscopic appearance and histological images of fresh human liver. (**c**) DNA quantification showing significant elimination of DNA in the decellularized cubes. (**d**) Comparison of the expression and distribution of several ECM proteins, namely collagen I, collagen III, collagen IV, fibronectin and Laminin, evaluated by immunohistochemistry showing consistency between decellularized cubes (top panel) and fresh samples (bottom panel). Data are expressed as mean ± s.d. ***p < 0.0001. Scale bars, 1 mm macroscopic images (**a**,**b)** or 200 μm top panel; a, b or 50 μm bottom panel; a, b or 100 μm (**d**). Biological replicates (n = 16) are performed for all samples.

Subsequently, the applied *g*-force was further increased to 45 *g* to minimize the time of exposure to detergents and to enhance the distribution of reagents within the tissue. Remarkably, the liver tissue cube turned translucent in just three hours (Fig. 1a) when compared to native tissue (FL) (Fig. 1b). H&E staining confirmed removal of nuclear materials while collagen and elastin filaments were preserved in the acellular liver tissue cube (ALTC) as showed by SR and Elastin Van Gieson (EVG) staining, respectively (Fig. 1a). Moreover, DNA quantification showed a significant reduction in the total amount of DNA after the procedure (p < 0.0001; Supplementary Fig. 1e), to below the threshold of 50ng/mg of tissue^16^. These observations suggested that by employing high shear stress, it is possible to obtain in a significantly reduced processing time, an ALTC with preserved collagen and elastin filaments while removing cellular material.

### ECM protein distribution and composition

Immunohistochemical analysis showed that the expression and distribution of key ECM components, namely collagen type I, collagen type III, collagen IV, fibronectin and laminin (Fig. 1d), were maintained in ALTCs compared to native liver tissue.

Furthermore, the composition of ECM proteins within the ALTCs was qualitatively investigated by proteomic analysis and compared to that of fresh liver tissue (Table 1). Notably, the main fibrillar and structural collagens (collagen alpha-1,-3,-6), as well as laminins, were preserved after decellularization. In addition, other ECM proteins were identified only within the acellular scaffold and were mainly represented by proteoglycans (lumican and mimecan) and glycoprotein (vitronectin) (Table 1).

**Table 1:** Identification of ECM proteins. Proteomic results for fresh liver samples and ALTCs confirming the preservation of the ECM proteins.

UniProt Entry Name	Protein	Gene	Organism	PE	SV	Protein Score
Fresh liver samples						
CO1A1_HUMAN	Collagen alpha-1(I) chain	COL1A1	Homo sapiens	1	5	97
CO3A1_HUMAN	Collagen alpha-1(III) chain	COL3A1	Homo sapiens	1	4	36
CO6A1_HUMAN	Collagen alpha-1(VI) chain	COL6A1	Homo sapiens	1	3	69
CO1A2_HUMAN	Collagen alpha-2(I) chain	COL1A2	Homo sapiens	1	7	112
CO6A3_HUMAN	Collagen alpha-3(VI) chain	COL6A3	Homo sapiens	1	5	131
FINC_HUMAN	Fibronectin	FN1	Homo sapiens	1	4	31
LMNB1_HUMAN	Lamin-B1	LMNB1	Homo sapiens	1	2	17
LMNA_HUMAN	Prelamin-A/C	LMNA	Homo sapiens	1	1	92
**Acellular liver tissue cubes (ALTCs) samples**						
CO1A1_HUMAN	Collagen alpha-1(I) chain	COL1A1	Homo sapiens	1	5	379
CO3A1_HUMAN	Collagen alpha-1(III) chain	COL3A1	Homo sapiens	1	4	59
CO6A1_HUMAN	Collagen alpha-1(VI) chain	COL6A1	Homo sapiens	1	3	204
CO1A2_HUMAN	Collagen alpha-2(I) chain	COL1A2	Homo sapiens	1	7	268
CO4A2_HUMAN	Collagen alpha-3(VI) chain	COL4A2	Homo sapiens	1	4	58
CO6A2_HUMAN	Collagen alpha-2(VI) chain	COL6A2	Homo sapiens	1	4	206
CO6A3_HUMAN	Collagen alpha-3(VI) chain	COL6A3	Homo sapiens	1	5	783
FINC_HUMAN	Fibronectin	FN1	Homo sapiens	1	4	171
LAMA5_HUMAN	Laminin subunit alpha-5	LAMA5	Homo sapiens	1	8	81
LAMB2_HUMAN	Laminin subunit beta-2	LAMB2	Homo sapiens	1	2	31
LUM_HUMAN	Lumican	LUM	Homo sapiens	1	2	28
MIME_HUMAN	Mimecan	OGN	Homo sapiens	1	1	85
VTNC_HUMAN	Vitronectin	VTN	Homo sapiens	1	1	105

Overall, this analysis confirmed the preservation of key ECM components in the acellular scaffold and suggested that the analysis of the cell-free scaffold may reveal the presence of ECM proteins which are masked by the cell-dependent protein background during proteomic analysis.

### 3D architecture and ultrastructure

**Figure 2:**
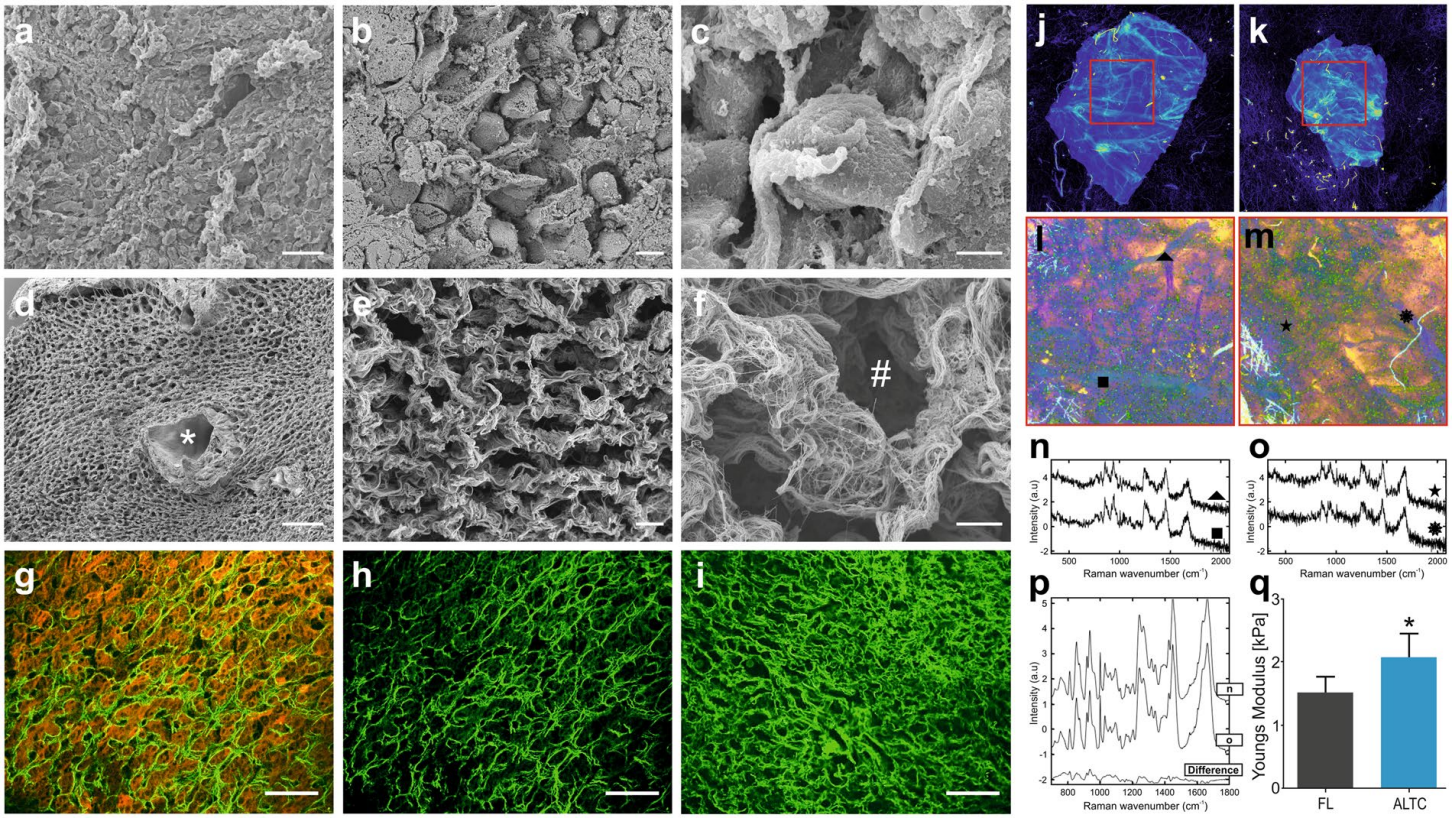
Confirmation of preservation of the micro-anatomy, biochemical and biomechanical properties of the ALTCs. SEM imaging of; (**a–c)**, fresh liver samples, and (**d–f)**, decellularized liver cubes showing the preservation of a portal tract (asterisks), collagen fibrils and hepatocyte pockets (octothorpe). Second harmonic generation analysis of fibrillar collagens structure (green) of (**g**), fresh liver samples with the presence of cells (red), (**h**), fresh liver samples with the subtraction of cells and (**i**), decellularized liver cubes. (**j,k**) Confocal auto-fluorescence microscopy showing preservation of the vascular trees in both samples. (**l,m**) Pseudo-colour images based on the first six components, highlighting the overall morphology of the tissue samples. (**n,o**) Raman spectra from different points on the cubes, showing similar spectra pattern to collagen. (**p**), Biochemical peaks of collagen proteins demonstrating no variability between the 2 decellularized cubes. (**q**) AFM comparison of tissue stiffness between fresh and decellularized liver cubes. Data are expressed as mean ± s.d. *, p < 0.05. Scale bars, 100 μm (**a,d)**, or 10 μm (**b,c,e,f**), or 100 μm (**g–i**). Biological replicates (n = 8) are performed for (**a–i)**, or (n = 3) for **q**.

Scanning electron microscopy (SEM) was used to compare fresh LTCs (Fig. 2a–c) with ALTC and to assess the quality of the resultant ECM micro-architecture after the decellularization procedure (Fig. 2d–f). SEM imaging revealed the maintenance of key hepatic features including the honeycomb-like arrangement and organized network of ECM fibrils associated with liver lobules (Fig. 2f). In addition, portal tracts were preserved after decellularization (Fig. 2d, *) as well as the size of cellular pockets within the parenchymal space which was in the region of 15–30 μm corresponding to the approximate size of a hepatocyte (Fig. 2f, #). Overall, these data confirm the preservation of the 3D liver microanatomy and ultrastructure following decellularization.

Visualisation of collagen type I expression and distribution was conducted via second harmonic generation (SHG) microscopy allowing the simultaneous visualisation of collagen type I and cell autofluorescence (Fig. 2g–i). The decellularized tissue (Fig. 2h–i) was characterized by the presence of more concentrated regions than the native tissue sections (Fig. 2g) due to the removal of cellular materials which outdistance the 3D organisation of collagen fibres. Regardless, the alignment and the distribution of collagen type I fibres in the decellularized tissue reflected the normal hepatic 3D architecture.

### Biochemical and biomechanical features of decellularized liver tissue

Intrinsic tissue auto-fluorescence confocal imaging (AF) and Raman spectroscopy (RS) were used for further biochemical analysis of the decellularised tissue. The results obtained on two different samples (derived from two independent scaffold preparations) (Fig. 2j,k) showed strong tissue auto-fluorescence emission in the wavelength range 450–480 nm when the excitation was performed at 405 nm, indicating the high content of collagen^31^. The AF images were also used to select 4 × 4 mm^2^ measurement areas for RS. Principal component analysis (PCA) was used to build spectral maps (Fig. 2l,m), highlighting the main spectral variance in the dataset (first six principal components, capturing 45.2% of variance, were combined). Figure 2n,o show typical Raman spectra selected at positions corresponding to strong fluorescence emission in Fig. 2j,k. The Raman spectra showed similar band pattern to Raman spectra of collagen type I reported previously^32^, including intense bands at 851 cm^−1^ and 950 cm^−1^ assigned to proline and hydroxyproline, 1004 cm^−1^ corresponding to phenylalanine, amide III bands at 1246 cm^−1^, 1271 cm^−1^, CH_2_ deformations at 1450 cm^−1^ and Amide I at 1660 cm^−1^. K-means clustering (k = 3) was then used to analyse the entire dataset. The centroid spectra for both samples are illustrated in Fig. 2p and confirmed the chemical similarity of the two samples. Furthermore, the analysis confirmed the lack of Raman bands typical of cellular material^33^.

Biomechanical characterisation of decellularized and native liver tissue was conducted with atomic force microscopy (AFM). Calculation of Young’s modulus from the AFM force curves demonstrated a significant difference (p < 0.05) between the stiffness of decellularized liver tissue and the native tissue (Fig. 2q). As previously described in other studies on biological scaffolds^34^, the Young’s modulus calculated for the decellularized liver tissue was 2.051 ± 0.39 kPa (SEM), i.e. approximately twice as great as that calculated for the native liver tissue (0.98 ± 0.14 kPa).

### Pro-angiogenic properties of acellular human liver scaffold

**Figure 3:**
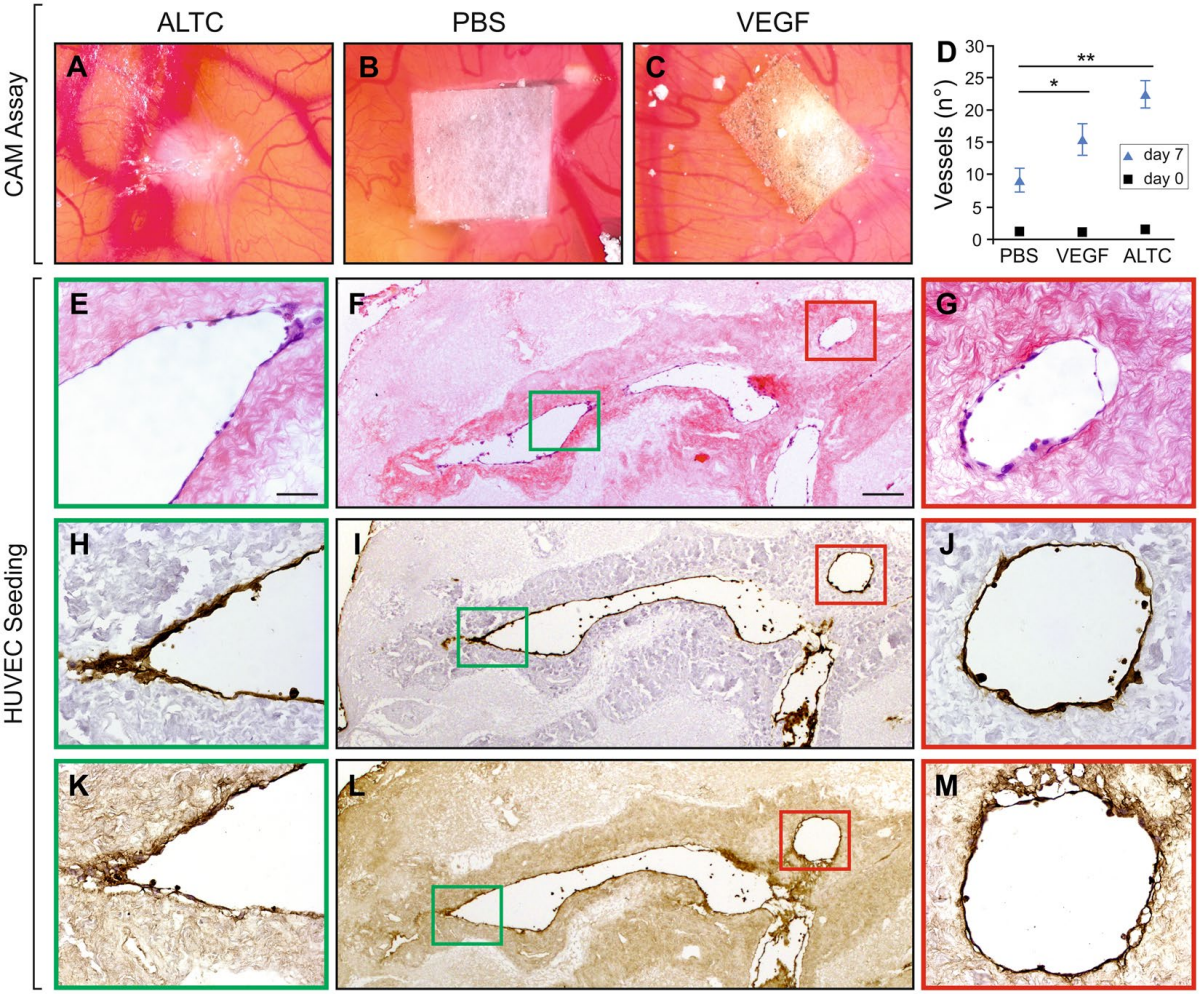
Neo-angiogenesis and re-endothelisation of ALTCs. CAM assay of (**A**), decellularized liver cube, (**B**), sponge soaked in PBS, and (**C**), sponge loaded with VEGF. (**D**) Quantification of observed vessels at 0 and 7 days, showing a significant difference between the ALTCs after 7 days when compared to the negative control (PBS). Recellularization of ALTCs with HUVECs after 7 days, characterized by positive staining with (**E–G**), H&E, (**H–J)**, CD31 and (**K–M)**, FVIII, confirming the migration and attachment of the endothelial cells to the lumen of the vessels and their functionality. Data are expressed as mean ± sem. *p < 0.05, **P < 0.005. Scale bars, 50 μm (**E,G,H,J,K,M**), or 200 μm (**F,I,L**). Biological replicates (n > 5) are performed for all samples.

The ability of ECM scaffolds to attract blood vessels and to promote neo-angiogenesis has been demonstrated in several studies^35–38^. This feature was investigated by employing the chicken egg chorioallantoic membrane (CAM) assay (Fig. 3A–D). Fragments of decellularized liver cubes were placed on the CAM system and observed macroscopically for 7 days. The ALTCs were well incorporated within the developing environment of the chicken egg CAM system, which was demonstrated by the attraction of blood vessels in a spoked-wheel pattern towards the scaffolds (Fig. 3A). Notably, the number of vessels growing towards the ALTCs was significantly higher than both negative control - the membrane loaded with PBS (Fig. 3D, p < 0.005) and positive control - pro-angiogenic cytokine VEGF-loaded membrane (Fig. 3C,D).

### Re-endothelization of acellular liver tissue cubes

The re-cellularization of intra-hepatic vessels by employing human umbilical vein endothelial cells (HUVECs) was then assessed. HUVECs were seeded into the ALTC and their presence/distribution was evaluated after 3 and 7 days of culture. Initially, endothelial cells were attached to the surface of the scaffold (3 days; data not shown) and after 7 days cells were uniformly lining the luminal surface of large vessels (Fig. 3E–G). In addition, the homogeneous expression of platelet endothelial cell adhesion molecule (PECAM-1) (Fig. 3H–J) and FVIII confirmed the maintenance of endothelial-like features (Fig. 3K–M).

### Bioengineering of human liver scaffolds with liver-specific pericytes

**Figure 4:**
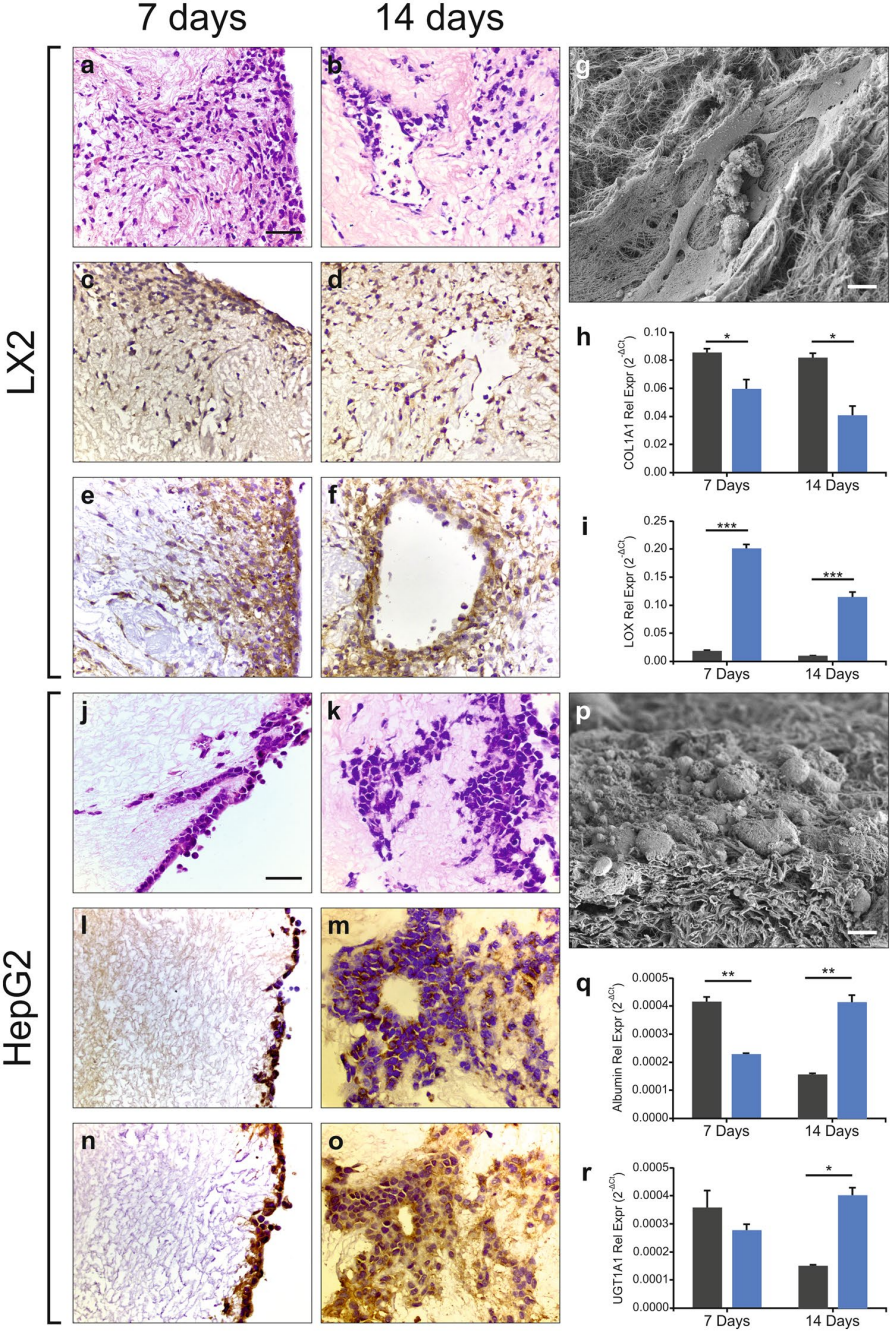
Biocompatibility of ALTCs with various cell lines. Reseeded HepG2 cells were positive for (**a,d)**, H&E, (**b,e)**, AFP and (**c,f)**, EPCAM staining at both 7 (left panel) and 14 days (right panel). At 7 days the cells appear to have only attached to the outer surface of the ALTCs, however, at 14 days, they were able to migrate and occupy the ALTC’s parenchymal space. (**g**) SEM analysis confirming cellular attachment of the HepG2s cells to the ALTCs at 14 days.Quantitative comparison of (**h)**, albumin and (**i**), UGT1A1 mRNA expressions of HepG2 grown on 2D plastic (black bar) and those reseeded on ALTCs (blue bar), show contrasting patterns after 7 and 14 days. Similarly, reseeded LX2 cells stained positive for (**j,n**), H&E, (**k,o**), PDGFB-β and (**l,p**), TFG-β at both 7 and 14 days. In contrast to HepG2, LX2 cells were able to migrate and occupy the ALTCs sinusoidal space at 7 days and were more abundant after 14 days. (**p**), SEM analysis confirming cellular attachment of the LX2 cells to the ALTCs at 14 days. Quantitative comparison of (**q**), COL1A1 and (**r)**, LOX mRNA expressions of LX2 cells grown on 2D plastic (black bar) and those reseeded n ALTCs blue bar), confirming significantly different patterns. Data are expressed as mean ± s.e.m *p < 0.05, **p < 0.005, ***p < 0.0001. Scale bars, 50 μm (**a–f**, **j–o)**, or 10 μm (**g,p**). Biological replicates (n > 4) are performed for all samples.

Acellular liver tissues were reseeded with liver-specific pericytes, i.e. hepatic stellate cells, and engineered tissues were evaluated after 7 and 14 days of *in vitro* culture. H&E staining demonstrated the progressive engraftment of LX2 cells, a human hepatic stellate cell line, into the scaffold after 14 days compared to 7 days (Fig. 4j,m). SEM analysis demonstrated that LX2 cells migrated within the decellularized sinusoidal space acquiring a definite myofibroblast-like cell phenotype (Fig. 4p). These findings were also confirmed with primary human hepatic stellate cells cultured up to 7 days which demonstrated cell engraftment and cell viability (Supplementary Fig. 3).

The myofibroblastic phenotype of the cells migrated into the scaffold was assessed by the expression of cellular markers typically expressed by hepatic stellate cells in their activated pro-fibrogenic phenotype^39^. The expression of key growth factor receptors such as platelet derived growth factor beta receptor (PDGFβ-R) (Fig. 4k,n) and transforming growth factor beta receptor (TGFβ-R) (Fig. 4l,o) was detected by immunohistochemistry. However, an interesting difference in the mRNA expression of two key pro-fibrogenic molecules, namely collagen type-1 and lysyl oxidase (LOX) was observed by comparing cells cultured in ALTCs versus standard 2D conditions. Indeed, collagen type-1A1 expression was significantly reduced in 3D culture (p < 0.05 at 7 days, and p < 0.005 at 14 days) (Fig. 4q), while LOX2 was significantly increased in cells cultured in 3D conditions when compared with 2D culture (p < 0.0001) (Fig. 4r).

### Bioengineering of human liver scaffolds with parenchymal liver cells

Next, liver tissue scaffolds were reseeded with HepG2, a human hepatoblastoma cell line reproducing many of the features of polarized human hepatocytes^40^ and largely employed as a cellular component for bioartificial livers^41^. After 7 days of seeding, HepG2 cells were found attached to the surface of the scaffold, while a more homogenous distribution within the scaffold was observed after 14 days, respectively (Fig. 4a,d). HepG2 cells were characterized by an epithelioid phenotype as shown by SEM and were diffusely spread and engrafted into the ECM scaffold (Fig. 4g). Immunohistochemical staining revealed positivity for alpha feto protein (AFP) at 7 days and the intensity was reduced after 14 days of incubation (Fig. 4b,e). In addition, cell adhesion within the acellular tissue was confirmed by the expression of the epithelial cell adhesion marker (EpCAM) at both 7 and 14 days (Fig. 4c,f). Moreover, the expression of human albumin and phase II metabolism enzyme UGT1A1 was evaluated in order to assess the metabolic activity of the engrafted cells. Quantitative RT-PCR revealed that albumin expression level was higher in the recellularized liver at 14 days when compared with a standard 2D culture, thus suggesting that the 3D acellular tissue was able to maintain the synthetic hepatocyte-like phenotype for a longer time period (Fig. 4h, p < 0.0001 versus 2D culture). This finding was also supported by a higher level of expression of phase II metabolism enzyme UGT1A1 at 14 days in the engineered tissue (Fig. 4i, p < 0.005 versus 2D culture).

**Figure 5:**
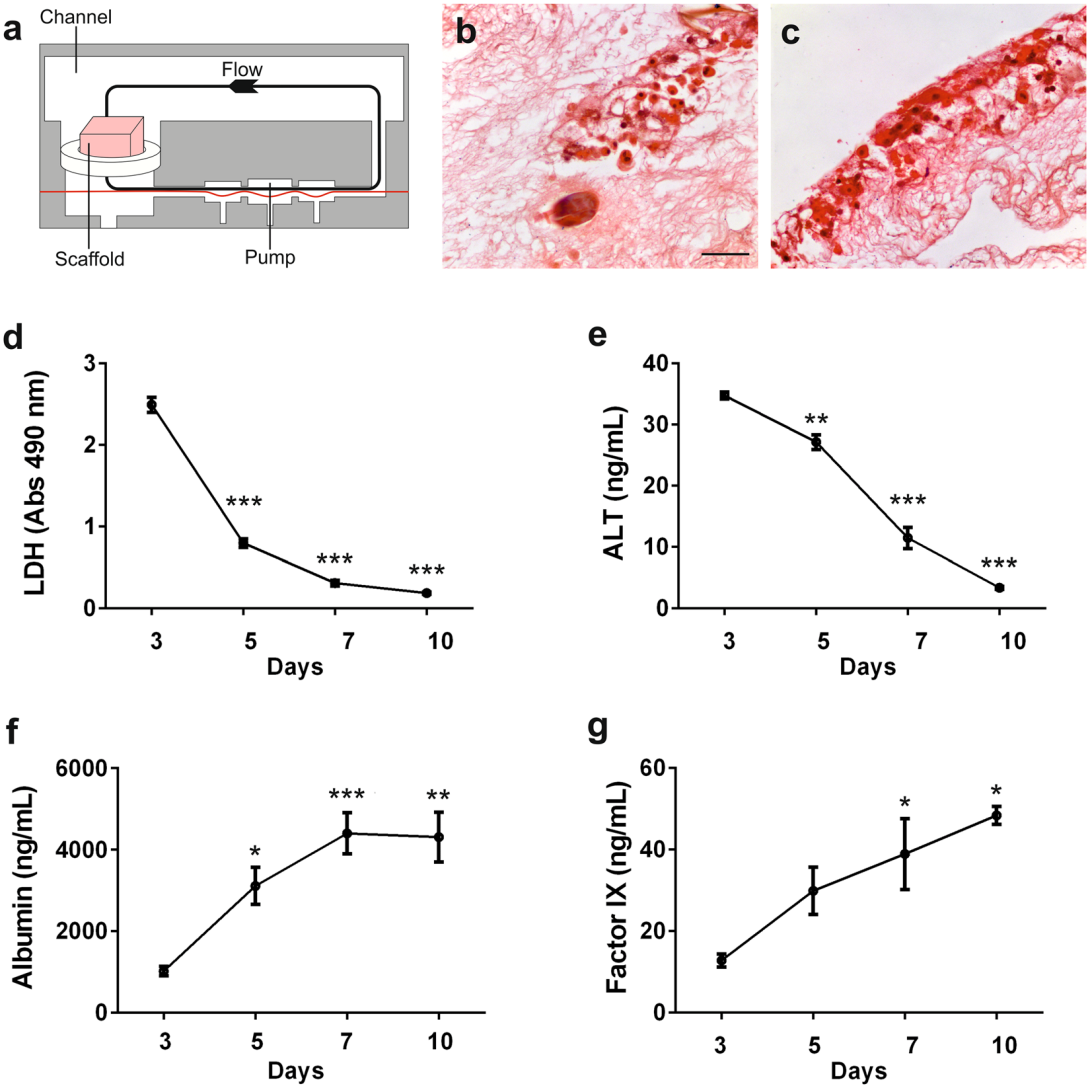
Biocompatibility of ALTCs with primary hepatocytes in a dynamic perfusion culture system.: Human primary hepatocytes cultures were established in ATLC using the LiverChip dynamic system for up to 10 days. (**a)** Schematic representation of ALTCs with cells in the perfusion system. H&E staining showing the attachment of primary hepatocytes in the, (**b)**, core and, (**c)**, outer surface of the ALTC after 10 days in culture. Hepatocytes supernatants were collected every 48 hours for measurement of lactate dehydrogenase (LDH), alanine aminotransferase (ALT), Albumin, and factor IX. Data are expressed as mean ± s.e.m. and analysed via one-way ANOVA with Dunnet’s multiple comparisons test, *p < 0.05, **p < 0.005, ***p < 0.0001. Scale bars, 50 μm. Biological replicates (n = 6) are performed for all samples.

Lastly, human liver scaffolds were reseeded with primary human hepatocytes by employing a dynamic perfusion system^42, 43^. This system was implemented in order to improve both cellular distribution within the scaffold and the oxygenation of engineered tissues (Fig. 5a). Primary human hepatocytes engrafted into the human liver scaffold under dynamic condition (Fig. 5b,c). Notably, during the first days of reseeding ALT and LDH increased because of the stabilization of the primary cells into the scaffolds. After day 3, these molecules were dramatically reduced while the metabolic and protein synthesis activities were increased up to day 10 of reseeding as shown by albumin and FIX secretion, respectively (Fig. 5d–g).

All together these findings demonstrated adequate cellular viability and function after scaffold engineering.

## Discussion

This work demonstrates that small scale human liver biological scaffolds can be derived by process of agitation-decellularization employing high shear stress (high g-force) which greatly reduces processing time. The resultant 3D-human liver scaffolds are characterized by the preservation of essential biochemical, physical and topographical properties and in addition provide an optimal platform for tissue bio-engineering employing different types of human liver cells.

The methodology based on agitation-decellularization was pioneered by Meezan and colleagues in ref. 23, who proposed this approach for studying the basement membrane microstructure. Subsequently, this methodology has been adapted to decellularize sections of different tissues, including xenogenic liver, by incubating the native tissue with different detergents without a precise definition of the mechanical forces involved in the process^24–30^.

The key contribution of the work herein presented is the development of a novel decellularization protocol based on high g-force oscillation leading to the successful removal of immunogenic cellular materials, while maintaining the ECM protein composition and 3D architecture. This achievement represents a key advance towards the use of wedge sections of normal human liver tissue obtained following surgical resections for the development of biological human liver ECM scaffolds. The resultant biomaterial can be engineered and employed as platform for human disease modelling and the assessment of drug efficacy/toxicity.

To this aim, a necessary step is represented by the ability of the decellularized tissue to be repopulated by organ-specific cell types. The present work provides solid evidence that human parenchymal and non-parenchymal cell are able to engraft, to grow and to express a functional phenotype within the human acellular liver tissue developed with the newly proposed protocol. Indeed, the expression of phenotype-specific markers for both human parenchymal and non-parenchymal liver cells indicated that the engrafted cells maintain differentiation and functionality. For example, HepG2 cells grown in 3D-scaffolds were able to maintain higher cellular differentiation features following prolonged culture when compared to the same cell preparation grown on standard 2D-plastic conditions.

Similarly, LX2 cells grown in 3D-scaffolds retained the phenotype of activated cells. Importantly, the mRNA expression of key pro-fibrogenic genes (collagen type-1 and LOX2) showed a remarkable difference with the same cell preparation grown on standard 2D-plastic conditions. These observations highlight the presence of remarkable differences in the biology of liver cells cultured in a liver 3D ECM scaffold when compared to standard 2D culture conditions on plastic dishes and definitely shed doubts on the accuracy and validity of the research for molecular targets of disease when in *vitro* studies are conducted on artificial 2D plastic systems.

Although small scale scaffolds cannot be perfused directly through the main vascular system, the ALTC maintain the vasculature structure and protein composition of the native tissue. This was confirmed by demonstrating the efficient re-endothelization obtained with human endothelial cells. Furthermore, the metabolic function and protein synthesis of engineered liver scaffolds with human primary hepatocytes reseeded under dynamic conditions were preserved.

Altogether the current results provide an innovative basis for further developments in the area of all human *in vitro* 3D-platforms for the study of disease pathophysiology, pharmacological target discovery, drug toxicity assessment and possibly micro-tissue transplant.

## Methods

### Source of human livers

All organs used for this study were retrieved through the NHSBT National Organ Retrieval Service and was coordinated by the UCL Tissue Access for Patient Benefit organisation (TAPb). The study was approved by the UCL Royal Free Biobank Ethical Review Committee (NRES Rec Reference: 11/WA/0077). Informed consent for research was confirmed via the NHSBT ODT organ retrieval pathway, and the project was also approved by the NHSBT Research Governance Committee. Donor livers were processed in accordance with the UCL Royal Free Biobank protocols under the Research Tissue Bank Human Tissue Act licence, prior to use in research. Before use for experimentation, discarded livers were histologically analysed, and those negative for fibrosis and steatosis were marked as “healthy” and used for this study.

### Decellularization Protocols

Human livers (n = 4) were first perfused with 1% PBS (Sigma-Aldrich, U.K) to eliminate blood and then frozen at −80 °C for a minimum of 24 h to assist with the initial destruction of the cellular membrane.

Afterwards, human livers were thawed at 4 °C overnight and cut into 125mm^3^ cubes (n > 1000) and stored again at −80 °C until future use.

Prior to decellularisation, liver tissue cubes (LTCs) were thawed in a water bath at 37 °C for 1 hour, followed by the addition of 1.2 ml of 1% PBS for 15 minutes. Once thawed the cubes were transferred into 2 ml safe-lock tubes (Eppendorf). A standardised 1.5 ml of each solution is added to its respected tube/protocol. The agitation protocols for the decellularization of the LTC is presented in Supplementary Table 1.

### Histology and immunostaining analysis

Sections (4 μm) were taken from formalin fixed paraffin embedded blocks and were dewaxed and rehydrated prior to staining (Xylene + IDA).

The histochemical stains were performed at room temperature as follows:

#### Haematoxylin and Eosin

Sections were treated with haematoxylin Harris’ formula (Leica Biosystems) for 10 minutes and then washed in tap water for 5 minutes. Next, the sections were stained with eosin (Leica Biosystems) for 3 minutes and then washed again with water. The sections were then dehydrated through IDA as quickly as possible and then placed in histology grade xylene until mounted.

#### Pico-Sirius Red

Sections were treated with freshly filtered pico-sirius red – F38 (R.A.Lamb; CI-35780) for 20 minutes. The sections were then dehydrated through IDA as quickly as possible and then placed in histology grade xylene until mounted.

#### Elastic Van Gieson

Sections were treated with 0.5% potassium permanganate for 5 minutes and washed thoroughly with distilled water. Next, they were treated with 1% oxalic acid for 1 minute, washed with distilled water followed by absolute alcohol. Sections were then stained with neat Miller’s Elastic - (R.A. Lamb; LAMB/080D) for 2 hours, washed thoroughly with 70% industrially methylated spirits (IMS) (Fisher Scientific) and then placed in tap water. The sections were checked under the microscope and, if necessary, differentiated in 0.5% acid-alcohol (1% HCl in 70% IDA aq.). As a final step, the sections were stained with van Gieson (Leica Biosystems) for 5 minutes.

Immunocytochemistry: sections stained with Collagen I, III, IV, fibronectin and laminin were performed at previously described by Mazza *et al*.^22^. Briefly, slides were pre-treated to retrieve the antigens by trypsinization for 30 minutes at 37 °C. Sections stained with alpha-fetoprotein (AFP), Factor VIII, TGF- β and CD31 were microwaved (640 W) for 5 minutes, 10 minutes, 15 minutes and 20 mins respectively and EPCAM and PDGF-β and pressure cooked for 3 minutes, in sodium citrate buffer (pH 6.0).

Sections were then blocked using a peroxidase solution for 5 minutes then incubated for 1 hour in the following primary antibodies; AFP (1:200; Dako, A0008), collagen I (1:200; Abcam, ab34710), collagen III (1:500; Abcam, ab7778), collagen IV (1:25; Dako, M0785), EPCAM (1:50; Leica Biosystems, NCL-ESA), (fibronectin (1:100; Millipore, MAB1937), laminin (1:200; Millipore, MAB1924), and Factor VIII (1:1000; Dako, A0082), PDGF-β (1:50; abcam, ab32570) and TGF-β (1:50; Santa Cruz, sc-146).

Primary antibodies were detected using the Novolink^TM^ kit (Novocastra RE7280-K). Sections were dehydrated, cleared and mounted and images were captured with an Axiocam IcC5.

### DNA quantification

Fresh and decellularised LTCs marked for DNA were weighed and if necessary, cut to be between 15 and 25 mg in mass. Two hundred μl of proteinase K solution was added to each and the samples were then placed into a heating block at 56 °C for at least 16 hrs or until they were completely lysed. DNA was then extracted using the QIAGEN DNAeasy Blood and Tissue Kit according to the manufacturer’s instructions. The extracted DNA was eluted in 200 µl of buffer AE and was quantified using a NanoDrop ND-2000 spectrophotometer.

### CAM assay

#### Chicken chorioallantoic membrane (CAM) angiogenic assay

To evaluate the angiogenic capability of the ALTC *in vivo* ^36, 44^, CAM assays were performed as previously described by Ahmadi *et al*.^45^. Briefly, prior to implantation, fertilized chicken eggs (Henry Stewart and Co.) were incubated at 37 °C at constant humidity for 8 days, followed by the extraction of 2 ml of albumin. 1 mm^3^ ALTCs (n = 19), sterile inert mesh loaded with PBS (n > 5) and 200 ng/mL VEGF (Sigma) loaded sterile mesh (n > 5), were placed on the CAM through a 3 cm diameter circular incision to the shell of the eggs. Eggs were allowed to incubate for a further 7 days at 37 °C at constant humidity. Scaffolds were photographed *in ovo* with a stereomicroscope equipped with a camera system (Leica) and blood vessels less than 10 mm in diameter that were converging towards the inserts were counted by blinded assessors (n = 34) at day 7. The mean of the counts was considered.

### Scanning Electron Microscopy (SEM)

Samples were fixed in 2.5% glutaraldehyde. Next, samples were washed 3 times with 1% PBS, placed in a 0.5% PBS solution containing 25% sucrose and 10% glycerol for 2 h and then snap frozen in liquid Nitrogen. Once frozen, samples were fractured and then placed back into a 0.5% PBS solution containing 25% sucrose and 10% glycerol at RT for 1 h. Once thawed, the samples were fixed with 1% Osmium tetroxide in 1% PBS at 3 °C for 1.5 h and then washed with deionized water (dH_2_O). Samples were then dehydrated through a series of dH_2_O and ethanol baths until finally reaching 100% ethanol. Next, samples were dried using carbon dioxide and firmly fixed onto an aluminum stub with the fractured side opposite to the stub. This was followed by the addition of a 2 nm thick layer of Gold/ Palladium using a Gatan ion beam coater. Images were recorded with a 7401 FEG scanning electron microscope (Jeol, USA).

### Second Harmonic Generation (SHG) and imaging

Both native tissue and decellularized liver scaffolds were cryoprotected and sections were then set in a mold and covered in OCT cold embedding media and frozen. The OCT blocks containing the liver tissue/scaffolds were cryosectioned to a thickness of 20 μm and mounted on glass slides. Prior to measurements, samples were thawed for 15 min in PBS.

All SHG images were obtained using a custom built multiphoton microscope incorporating an upright confocal microscope (SP5, Leica) and a mode-locked Ti: Sapphire Laser (Mai Tai, Newport Spectra-Physics). Images of the SHG signal from collagen I were collected using an 820 nm excitation with SHG signal obtained with a 414/46 nm bandpass filter and multiphoton autofluorescence signal obtained with a 525/40 nm bandpass filter. A 25X, 0.95 NA water-immersion objective (Leica) was used to deliver the excitation signal and to collect the SHG emission signal from the sample. Images with a 600 μm × 600 μm field of view were obtained with 2048 pixel resolution and a line rate of 10 Hz giving a pixel resolution of $\sim$0.3 μm with 3X averaging on each acquisition to reduce the effect of noise.

### Atomic Force Microscopy (AFM)

#### AFM sample preparation

Tissue samples for AFM measurement were prepared by taking tissue slices from a cube of liver tissue. Thick tissue slices were cut from the tissue cube using a scalpel, under liquid conditions and were kept in PBS at room temperature before attachment to a petri dish for analysis within 30 minutes. A slice of tissue measuring 5mm × 2 mm × 2 mm was attached to a petri dish using two droplets of Cyanoacrylate adhesive, applied with a 10 μl pipette tip, placed at the extremities of the sample. After tissue slice attachment (1–2 min) the slice was immersed in PBS in order for the AFM measurements to be conducted within a 2 h time period. In the case of tissue scaffold samples, scaffold cubes were removed from storage in PBS at 4 °C and excess liquid removed with a tissue prior to attachment. As previously described for the tissue slices, tissue scaffold cubes were attached to petri dishes with two droplets of adhesive at the extremities. After attachment (1–2 min) scaffolds were immersed in PBS and imaged within 2 h.

#### AFM Measurements

Measurements of the tissue slices and scaffold cubes have been conducted on a JPK Nanowizard-1 (JPK Instruments) operating in force spectroscopy mode, mounted on an inverted optical microscope (IX-81, Olympus). AFM pyramidal cantilevers (MLCT, Bruker) with a spring constant of 0.07 N/m were used with a 35 μm glass bead attached to cantilever tip. Prior to measurements with the adapted cantilevers, their sensitivity was calculated by measuring the slope of the force-distance curve in the AFM software on an empty region of the petri dish. For indentation tests on the sample, the cantilever was aligned over regions in the middle of the samples using the optical microscope. For each sample 30–50 force curves were acquired in 6–10 different 100 μm regions, this arrangement allowed force-curves to be acquired in locations at least 50–100 μm apart. Force-curve acquisition was carried out with an approach speed of 5 μm/s and a maximum set force of 1.5 nN. Elastic moduli were calculated from the force-distance curves by fitting the contact region of the approach curve with the Hertz contact model, using the AFM software.

### Raman Spectroscopy

Raman spectra were collected using an inverted optical microscope (Eclipse Ti, Nikon) equipped with a 63x/ 1.2NA oil immersion objective. A 785 nm excitation laser, with the power of 50 mW at the sample, was used for excitation. The Raman back-scattered light was collected by the same objective and collimated towards an optical spectrometer (RiverD International) equipped with a deep-depletion back-illuminated CCD (Andor Technology, UK). Raman maps were measured by raster-scanning the A movable stage (Prior Pro-scan ii) is equipped to allow spectral imaging. For measurements, the samples were placed on a 1 mm thick quartz window. Raman spectral mapping were recorded for tissue areas of 4 × 4 mm with a step size of 20 μm. The integration time at each pixel was 0.5 seconds. Auto-fluorescence images of the tissue samples were recorded for the same areas using a confocal scanner (C2, Nikon) integrated to the Raman microscope. The excitation was based on a laser at 405 nm (10 mW) and detection in the range 450–480 nm.

### Proteomic

The proteomic analysis was performed on formalin-fixed samples and 5 μm slides were removed by scalpel cleavage and analyzed as previously described by Rodriguez *et al*.^46^. Following extraction into 10 mM Tris/1 mM EDTA/0.002% Zwittergent buffer (99 °C, 1.5 h) and sonication (1 h), samples were trypsinised (1.5 mg w/v) overnight at 37 °C and then reduced with dithiothreitol (50 µg) at 99 °C for 5 minutes. Digests were run on a nanoACQUITY™ UPLC system (Waters Ltd., Elstree, Hertfordshire, WD6 3SZ) coupled to an Orbitrap Velos Mass Spectrometer (Thermo Electron, Bremen, Germany. MS data files were analysed using Mascot^47^.

### Cell Culture

LX2 cells were maintained in Iscove’s Modified DMEM supplemented with 2 mM/L-glutamine, 0.1 mM/L non-essential amino acids, 1.0 mM/L sodium pyruvate and 20% foetal bovine serum (FBS). HepG2 (ATCC® HTB-52™, VA, USA) were maintained in Eagle’s Minimum Essential medium (EMEM), supplemented with Glutamax, 0.1 mM/L non-essential amino acids, 1.0 mM/L sodium pyruvate and 10% FBS. Human umbilical vein endothelial cells (HUVEC pooled; PromoCell) were cultured in low-serum (2% V/V) ECGM with a supplemental mix (PromoCell) and used between passage 3 and 5. All cells were sub-cultured every 3 days at a split ration of 1:3.

Primary Human HSCs (hHSC) were isolated from wedge sections of liver tissue, obtained from patients undergoing surgery at the Royal Free Hospital after giving informed consent (EC01.14-RF). Cells were isolated according to Mederacke *et al*.^48^, with modifications for human liver^49^.

The obtained HSCs were cultured in IMDM supplemented with 20% FBS, glutamine, nonessential amino acids 1X, 1.0 mM sodium pyruvate, 1X antibiotic-antimycotic (all Life Technologies), hereafter referred to as complete HSC medium. Experiments described in this study were performed on hHSC of at least three independent cell preparations, between passage 3 and 8.

All cells were maintained under standard conditions at 37 °C in a humidified incubator containing 5% CO_2_.

### Reseeding with human liver cells under static conditions

To prepare decellularised tissue for cell culture, scaffolds were sterilised using 1.5 ml of 0.1% peracetic acid (Sigma-Aldrich) in 4% ethanol (Fisher Chemical) for 45 minutes in an orbital shaker (Staurt) at 700 rpm. This was followed by replacing the solution with sterile 1X HBSS (Thermofisher Scientific) for 15 minutes on an orbital shaker. The sterile scaffolds were then placed in culture media 24 hours prior to the additions of cells [day −1].

For LX2, HepG2 and primary cells, 2 million cells in 20 µl were released on top of each ALTC. For HUVEC cells, 1.4 million cells in 20 µl were released on top of each ALTC Following cell seeding; samples were kept for 2 hours at 37 °C in a humidified incubator containing 5% CO_2_. This was followed by the addition of culture medium. On the following day, scaffolds were transferred to individual wells and fresh culture medium was added. Thereafter, wells and fresh media were changed every 3 days. Experiments were stopped at days 7 or 14 following seeding for LX2, HepG2 and primary cells and at days 3 or 7 for HUVEC cells.

### 3D dynamic culture of primary hepatocytes in the LiverChip™ system

Primary hepatocytes 3D-cultures were established using the LiverChip™ (CN-Bio Innovations) dynamic culture system^42, 43^. Cryopreserved human hepatocytes (HMCPTS, ThermoFisher Scientific) were cultivated according to the supplier’s protocol. In brief, hepatocytes were quickly thawed in a water bath at 37 °C, transferred to a 50 mL tube containing 40 mL pre-warmed recovery medium (CHRM®, ThermoFisher Scientific), and centrifuged at 100 × g for 10 min at room temperature. The resulting cell pellet was resuspended in plating medium, and viable cells were counted by trypan blue exclusion. The plating medium was Williams E medium supplemented with 5% FBS, 1 µM dexamethasone, 1% penicillin/streptomycin, 4 μg/mL insulin, 2 mM GlutaMax, 15 mM HEPES, pH 7.4. Human liver scaffolds were inserted inside the culture well and held in place using a modified retaining ring. Cells were seeded in the LiverChip™ plates at a density of 0.6 × 106 hepatocytes/scaffold in a volume of 1.4 mL. Hepatocytes were allowed to attach on the scaffold in a 5% CO2-incubator at 37 °C for 24 h with a down flow of 1 µL/second. After 24 hours, cultures were switched to maintenance medium, which was replaced every 48 hours for the duration of the experiment. The maintenance medium composition was as follows: Williams E medium supplemented with 0.1 μM dexamethasone, 0.5% penicillin/streptomycin, 6.25 μg/mL insulin, 6.25 μg/mL transferrin, 6.25 μg/mL selenous acid, 1.25 mg/mL bovine serum albumin, 5.35 μg/mL linoleic acid, 2 mM Glutamax, 15 mM HEPES buffer, and 1X Hepextend supplement (Gibco). Medium was replaced every 48 hours and used to measure, with respective kits, LDH activity (Promega), secreted levels of albumin (Abcam), factor IX (Abcam), and alanine transaminase (ALT, Antibodies Online).

### RNA extraction and qRT-PCR

Total RNA was extracted from 2D and 3D cultures using TRIzol reagent and RNeasy Universal Mini Kit (Qiagen). One microgram of total RNA was reverse transcribed with random primers and MultiScribe RT enzyme (Applied Biosystems).

Gene expression was measured using Taqman gene expression assays with the Applied Biosystems® 7500 Real-Time PCR system (Supplementary Table 2).

Expression levels for each gene were calculated using the delta Ct method^50^ and normalized to the ones of GAPDH as the reference gene. Graphs depict averages ± SEM of the relative gene expression data (n = 3 or 4/group). Statistical analyses were performed with ordinary two-way ANOVA with Bonferroni’s multiple comparisons test, using Prism GraphPad software.

### Statistical analysis

Results were expressed as mean ± s.d. All data was analysed with ANOVA or Student’s t-test. Two-tailed p values less than 0.05 were considered statistically significant.

## Electronic supplementary material

Supplementary Movie 1

Supplementary Figures
