# Supplementary material for: Rapid production of human liver scaffolds for functional tissue engineering by high shear stress oscillation-decellularization
Source: Sci Rep. 2017 Jul 17;7:5534. doi: 10.1038/s41598-017-05134-1 (PMC5514140; doi:10.1038/s41598-017-05134-1)
Supplement: Supplementary file 2 — Supplementary Figures [file 41598_2017_5134_MOESM2_ESM.doc]

**Rapid production of human liver scaffolds for functional tissue engineering by high shear stress oscillation-decellularization**

Giuseppe Mazza^1*^, Walid Al-Akkad^1^, Andrea Telese^1^, Lisa Longato^1^, Luca Urbani^2^, Benjamin Robinson^3^, Andrew Hall^1^, Kenny Kong^4^, Luca Frenguelli^1^, Giusi Marrone^1^, Oliver Willacy^1^, Mohsen Shaeri^5^, Alan Burns^2,6^, Massimo Malago’^1^, Janet Gilbertson^7^, Nigel Rendell^7^, Kevin Moore^1^, David Hughes^5^, Ioan Notingher^4^, Gavin Jell^8^, Armando Del Rio Hernandez^3^, Paolo De Coppi^2^, Krista Rombouts^1^and Massimo Pinzani^1^.

^1^UCL Institute for Liver and Digestive Health, Royal Free Hospital. University College London, London UK.

^2^Stem Cells and Regenerative Medicine Section, Developmental Biology and Cancer Programme, UCL Great Ormond Street Institute for Child Health. University College London, London UK.

^3^Department of Bioengineering, Cellular and Molecular Biomechanics. Imperial College, London UK.

^4^ School of Physics and Astronomy. University of Nottingham, Nottingham UK.

^5^CN Bio Innovations Limited. BioPark Hertfordshire, Broadwater Road, Welwyn Garden City, Hertfordshire UK

^6^Department of Clinical Genetics, Erasmus Medical Centre. Rotterdam Netherlands

^7^Wolfson Drug Discovery Unit, Centre for Amyloidosis and Acute Phase Proteins, Royal Free Hospital. University College London, London UK.

^8^Center for Nanotechnology and Regenerative Medicine, Division of Surgery and Interventional Science. University College London, London UK.

Address for Correspondence:

^*^Giuseppe Mazza, University College London (UCL) Institute for Liver and Digestive Health, Royal Free Hospital, Rowland Hill Street, London NW3 2PF, United Kingdom.

Email: [giuseppe.mazza.12@ucl.ac.uk](mailto:giuseppe.mazza.12@ucl.ac.uk)

Keywords: human liver, agitation-decellularization, high frequency oscillation, tissue engineering, human hepatic cells, dynami


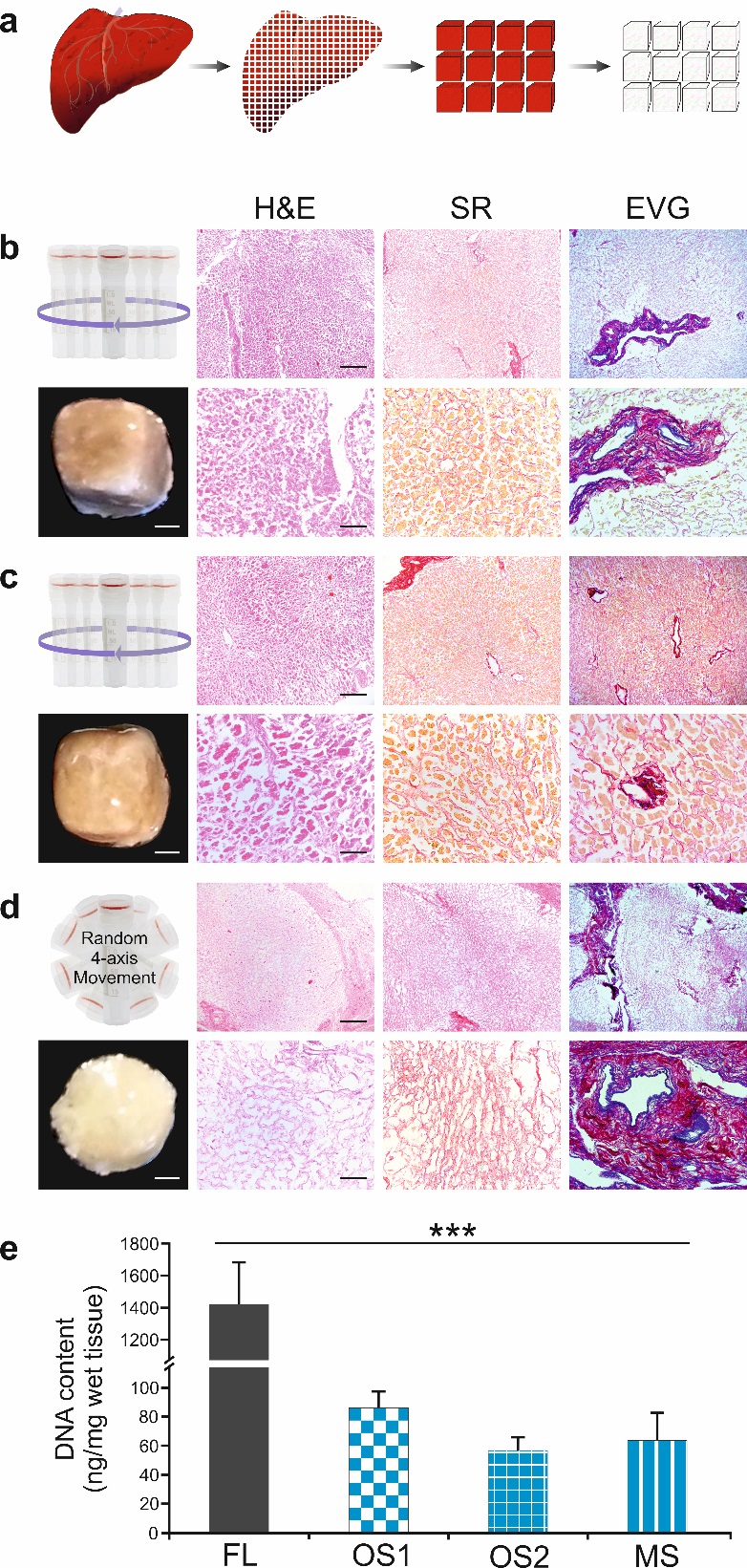


**Supplementary Figure 1 | Protocol optimization. a,** Representative schematic overview of human livers during the decellularization process. Macroscopic appearance and histological analysis after decellularization using an orbital shaker after **b,** 8 days and **c,** 16 days, showing elimination of nuclear material (blue; H&E) and preservation of elastin (blue/black; EVG) but failure to eliminate cellular material (yellow; SR). **d,** Macroscopic appearance and histological images of decellularized liver cubes using a magnetic stirrer after 8 days, showing elimination of nuclear (blue; H&E) and cellular material (yellow; SR) and preservation of collagen (red; SR) and elastin (blue/black; EVG). **e,** DNA quantification showing significant elimination of DNA in all decellularization protocols, although all protocols were above the desired quantity of 50ng/mg. Data are expressed as mean ± s.d. ***, p<0.0001. Scale bars, 1 mm macroscopic images **b, c, d** or 200 μm **top panel; b, c, d** or 50 μm **bottom panel; b, c, d.** Biological replicates (n>3) are performed for all samples.


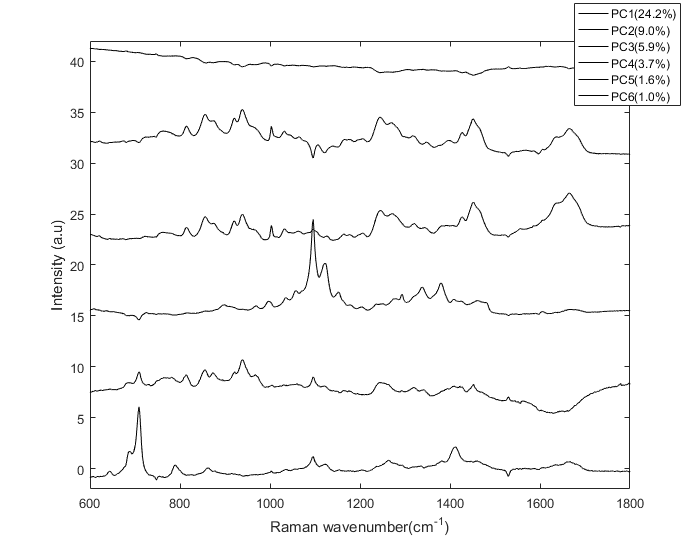
 **Supplementary Figure 2 | Plot of the first six components with their associated variance.** Principle component analysis (PCA) of the Raman spectra obtained from the liver scaffolds.


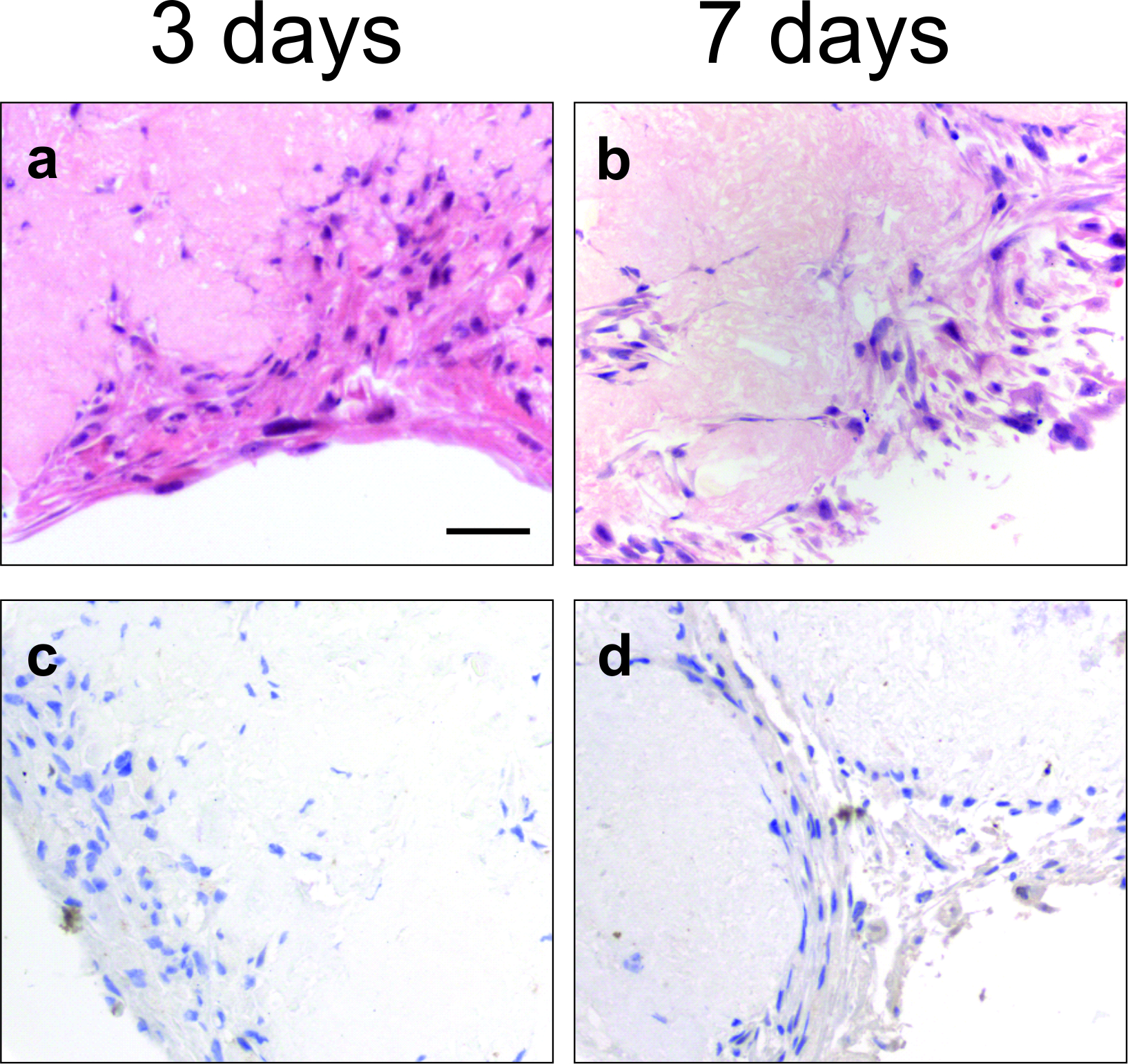


**Supplementary Figure 3 | Biocompatibility of ALTCs with human primary hepatic stellate cells.**

H&E staining showing the attachment of primary hepatic stellate cells at 3 days, a, and 7 days, b, of static culture conditions. To test the presence of cell apoptosis, IHC staining against ASP175 was performed demonstrating the absence of positive ASP175 staining at 3, **c**, and 7 days, **d**. Scale bar, 50 μm **a, b, c, d.** Biological replicates (n>3).

| **Low G-Force** | | | | |
| --- | --- | --- | --- | --- |
| **Step** | **Solution** | | | **Time (hours)** |
| 1 | Deionised Water | | | 24 |
| 2 | PBS 1X | | | 0.5 |
| 3 | Reagent Mixture | | | 5.5 |
| 4 | PBS 1X | | | 0.5 |
| 5 | DNase Solution | | | 3 |
| 6 | PBS 1X | | | 0.5 |
| - Repeat steps (1-6) 4 times for protocols OS1 and MS - Repeat steps (1-6) 8 times for protocol OS2 | | | | |
| **High G-Force** | | | | |
| **Step** | **Solution** | **Time (minutes)** | **Repetitions** | |
| 1 | Deionised Water | 2 | 5-20 | |
| 2 | Reagent Mixture | 2 | 1 | |
| 3 | Reagent Mixture | 4 | 2 | |
| 4 | Hypertonic Saline 9% | 2 | 5 | |
| 5 | Deionised Water | 2 | 5 | |
| 6 | Reagent Mixture | 2 | 1 | |
| 7 | Reagent Mixture | 4 | 2 | |
| 8 | PBS 1X | 5 | 3 | |
| - If necessary, repeat steps 5-8 | | | | |

**Supplementary Table 1 | Decellularization protocols.** Using low G-force and using high G-force. The following abbreviations were used: orbital shaker (OS), magnetic stirrer (MS), distilled Water (dH2O), TX100 (Triton X100), SDS (sodium dodecyl sulfate), SDC (Sodium Deoxyxholate), DNAse, A-A 5% (Antibiotic and Antimycotic), PAA (paracetic acid) and EtOH (ethanol).

| **Gene Name** | **Gene Abbreviation** | **Taqman Gene Assay No.** |
| --- | --- | --- |
| Glyceraldehyde-3-phosphate dehydrogenase | GAPDH | Hs02758991_g1 |
| Collagen Type 1, alpha 1 chain | Col1A1 | Hs00164004_m1 |
| Transforming growth factor, beta 1 | TGFβ1 | Hs00998133_m1 |
| Lysyl oxidase | LOX | Hs00942480_m1 |
| Integrin, alpha 6 | ITGA6 | Hs01041011_m1 |
| Albumin | ALB | Hs00609411_m1 |

**Supplementary Table 2 | Applied Biosystems Taqman Gene Expression Assay.**

Video uploaded separately.

**Supplementary Movie 1 |** Close up showing decellularization of human liver cube at normal speed and slow motion
